# Multiple Myeloma: Possible Cure from the Sea

**DOI:** 10.3390/cancers14122965

**Published:** 2022-06-16

**Authors:** Anita Capalbo, Chiara Lauritano

**Affiliations:** Stazione Zoologica Anton Dohrn, Ecosustainable Marine Biotechnology Department, Via Ammiraglio Ferdinando Acton, 80133 Napoli, Italy; anita.capalbo@szn.it

**Keywords:** multiple myeloma, marine natural product, cancer, bioactive compounds, marine organism

## Abstract

**Simple Summary:**

Multiple myeloma (MM) is a complex white blood cell (plasma cell, PC) cancer. The aetiology of MM is still unknown, and it is still an incurable disease despite efforts by the scientific community. The high level of PC genetic heterogeneity renders MM a complex puzzle to be solved. Combinations of drugs are generally used to treat MM patients, with a general increase in overall survival. Relapsed and refractory MM patients are the generation of patients who resist or do not respond to first-line therapy and need additional treatments. Exploring new sources, such as marine organisms, for drug discovery is fundamental to fighting MM. Various studies have shown that marine natural products (MNPs) might have antiproliferative and cancer-specific cytotoxic properties, giving MNPs a pivotal role in anticancer drug discovery. This review recaps updated frontline treatment options, including new ones developed from MNP research.

**Abstract:**

Multiple myeloma (MM) is a blood cancer that occurs in the plasma cells (PCs), a type of white blood cell. Despite the progress of several current treatments that prolong the overall patient’s survival, most MM cases are incurable. For this reason, many efforts have been undertaken by the scientific community in the search for new treatments. BLENREP^TM^ and Aplidin^®^ are two marine-derived drugs currently in use for MM. In addition, other natural products have been identified from marine organisms, tested for their possible anticancer properties, and are in preclinical or clinical trials for MM, including cytarabine, a compound in use for leukaemia treatment. Between the most successful marine compounds in fighting MM, there are molecules with specific targets, such as the elongation factor 1-alpha 2 and proteasome inhibitors, and compounds conjugated with antibodies that recognise specific cell types and direct the drug to the correct cell target. Active compounds belong to different chemical classes, from cyclic peptides to alkaloids, highlighting the importance of screening the plethora of compounds produced by marine organisms. In this review, we summarise the current state of art of MM therapies focusing on the marine natural product emerging roles.

## 1. Introduction

**Figure 1:**
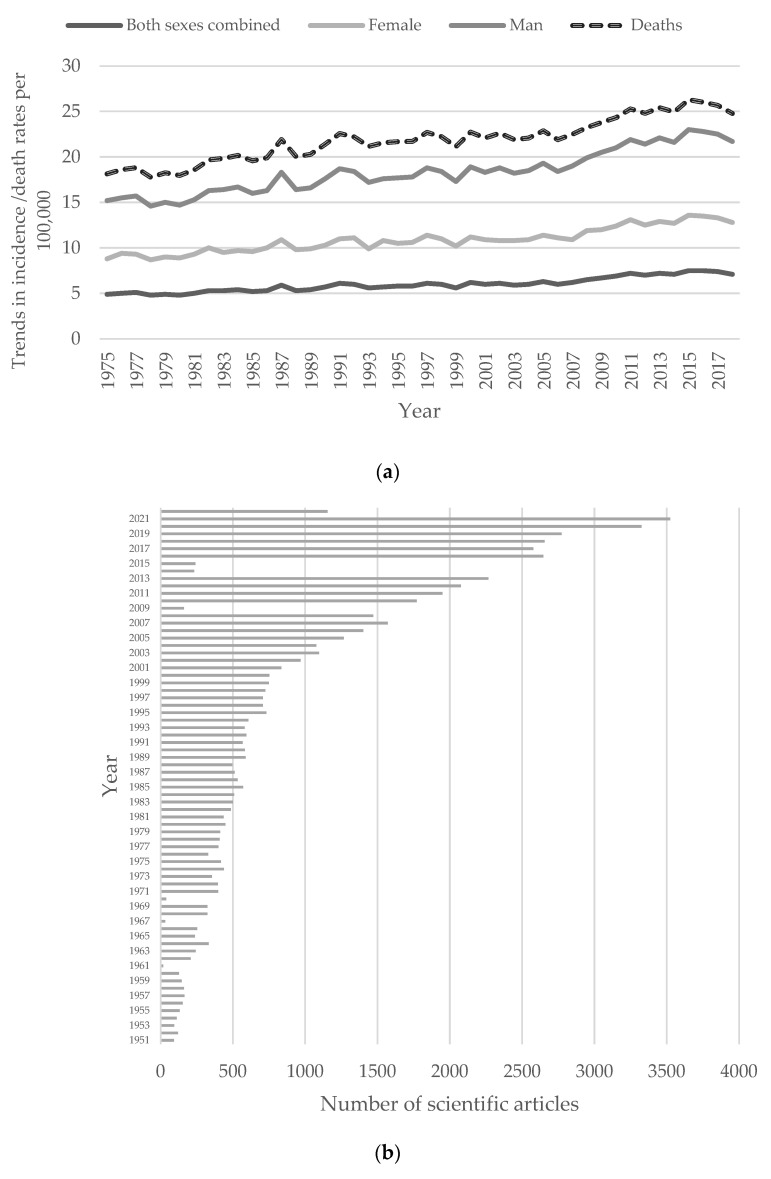
Multiple myeloma statistics. (**a**) Multiple myeloma trends in incidence or death rates. Data sources: Surveillance, Epidemiology, and End Results (SEER) 9 registries, National Cancer Institute, 2021© Copyright American Cancer Society, 2018; (**b**) Scientific literature on multiple myeloma over the last few decades. Adapted from data source: PubMed. Search query: “multiple myeloma”.

Myeloma is a cancer of the white blood cells called plasma cells (PCs) that, together with other immune system cells, plays an essential role in producing antibodies against exogenous pathogens. The main clinical symptoms are hypercalcemia, renal failure, anaemia, and bone lesions, also known as CRAB features [1,2]. Myeloma accounts for about 10% of all haematological malignancies [3,4]. According to the American Society of Cancer, trends in incidence and death rates are slightly decreasing (1975–2018) (Figure 1a). Nevertheless, estimated new cases in 2022 are 34,470, with 12,640 estimated deaths [5]. The decrease in the death rate trend correlates to developing different therapeutic strategies, increasing overall survival. The 5-year relative survival percentages expressed as the percentage of MM patients alive five years after the diagnosis, normalised on the percentage of the population, increased from 26.29% in 1975 to 59.05% in 2014 [6]. The risk of developing the disease is age-dependent, with increases in the population aged 65 to 74 [6]. Men are more likely to develop MM than women (Figure 1a). The aetiology of multiple myeloma is still not known. Nevertheless, scientific myeloma papers soared from a few articles in the 1950s to thousands in 2021, leading to a massive increase in fundamental insights into the molecular mechanisms and treatment of myeloma (Figure 1b). These data were obtained by searching the available literature on PubMed using “multiple myeloma” as a search filter.

The journey of normal PCs starts in the bone marrow (BM), where immature B cells move from the BM to lymphoid tissue such as the spleen. In their journey, they are exposed to exogenous material, typically germs (bacteria, viruses, etc.). After exposure to a single germ stimulus, B cells multiply into different clones. A part of them differentiates into PCs, and the other part remains as memory B cells for years. After differentiation, PCs return to the BM where they can stay for years in a quiescence status, remembering the specific germ that they met in their predifferentiation status. If reinfection occurs, they are activated and start clonal expansion, producing huge amounts of the same antibody needed to fight that specific germ. In this way, they can efficiently and rapidly fight the infection. In myeloma cancer, immature B cells with damaged DNA translocate into the lymphoid tissues. After exposure to a stimulus, they multiply and differentiate into PCs as in the normal condition. The disease starts when the damaged DNA of the generated PCs is not repaired. Nothing seems to be wrong in the system, and the generated PCs come back to the BM as usual. Once PCs start to multiply, huge amounts of a monoclonal antibody named paraprotein are secreted without control due to mechanisms that are still not completely clear [7]. Paraprotein or M-protein is a monoclonal antibody (MAb) that is useless to fight infections. It is considered to be one of the most important biomarkers for diagnosing monoclonal gammopathies of undetermined significance disease (MGUS). MGUS can evolve into smouldering multiple myeloma and then into multiple myeloma [3].

The high complexity of myeloma cancer gives rise to the clonal expansion of cancerogenic PC, which generates clones with different genetic features. In this way, pools of PC clones, different from each other, are generated. The clear and most harmful consequence of this genetic PC heterogeneity is that specific therapy can kill distinct pools of clones. In contrast, others are refractory to treatment and continue to grow. At the same time, clones affected by the therapy can develop resistance to treatments, leading to relapsed refractory multiple myeloma (RRMM). Clinicians are globally trying to overcome relapse and refractory cases by using several therapeutic strategies that differ regarding the used compound, timing, and duration [8]. Initial genomic studies of this particular cancer revealed how complex it is to identify a clear putative causative genome defect. Without a clear and homogeneous genetic landscape for myeloma, the correlation of genotype, phenotype, and therapy is a significant challenge [9]. What appears to be fairly consolidated by genome sequencing analysis is that patients show multiple subclones with a high level of heterogeneity [10,11]. The best-known representative genetic defects in myeloma can be classified into four main conditions: (1) switching of genetic material between different pairs of chromosomes (the most common is DNA translocation between 11 and 14 chromosomes); (2) the deletion of part of a chromosome or the addition of extra DNA within the chromosome; (3) hyperdiploidy; and (4) frequent point mutation affecting the DNA sequence (KRAS, NRAS, BRAF, and others) [10,11]. This pronounced genetic heterogeneity impairs and slows down the finding of efficient therapies.

## 2. Current Treatments

**Figure 2:**
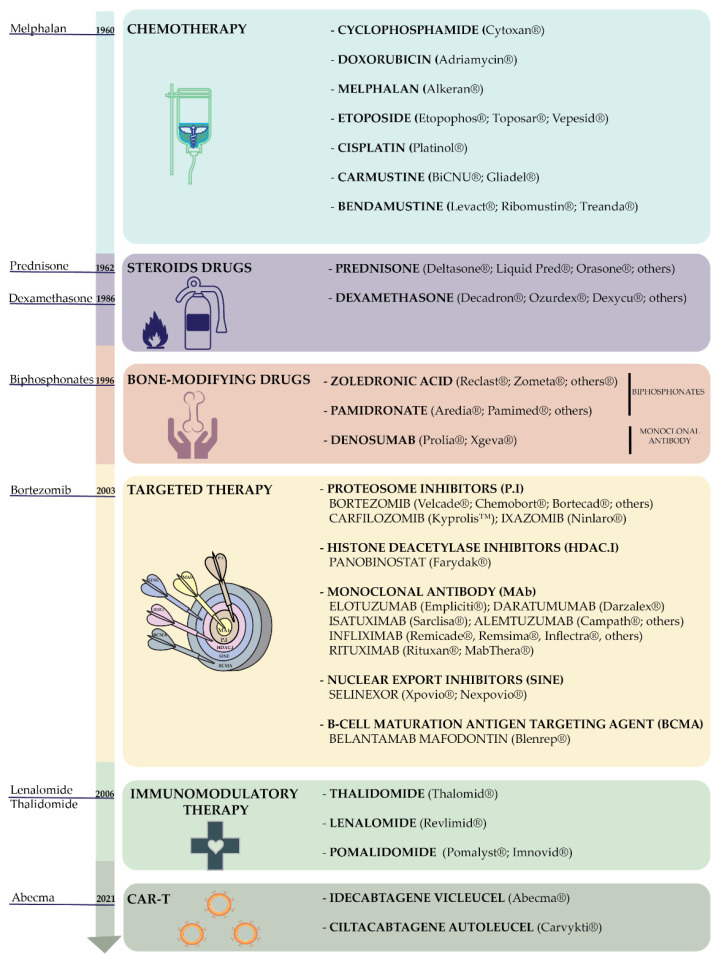
Schematic representation and positioning of pioneering drugs for treating multiple myeloma over the years (arrow). For each typology of drugs, the most representative compounds with their corresponding commercial names are reported in the specific boxes.

Frontline therapy includes high-dose chemotherapy or a combination of different drugs, discussed below, followed by autologous stem-cell transplantation when it is possible [12]. Myeloma therapies can be classified using the different molecular class strategies to which they belong, such as chemotherapy (kills fast-growing cells), immunomodulatory therapy (stimulates the immune system), steroids (reduce inflammation), targeted therapy that takes advantage of targeting a specific cancer gene or protein, bone-modifying drugs (which reduce osteoclast activity), vaccines (to stimulate the immune system) [13,14], and CAR-T technology (also known as chimeric antigen receptor T-cell therapies) [15]. Examples of targeted therapy are monoclonal antibodies that specifically recognise myeloma cells, thus activating the immune response to kill them [16]; monoclonal antibodies and a toxin, which recognise and anchor cancer antigens onto myeloma cells, thus releasing the toxin into the cancer cells and killing them [16]; nuclear export inhibitors (which block the nuclear export of RNA and messenger proteins, leading to cancer cell death) [17]; histone deacetylase inhibitors (the regulation of cancer differentiation and progression due to transcription regulation via the inhibition of histone deacetylase [18]); B-cell maturation antigen targeting agents (conjugated monoclonal antibodies and toxin); and proteasome inhibitors (block myeloma cell proteosome causing their death due to the accumulation of the internal overproduction of paraprotein that cannot be either recycled or deleted [19]. The historical introduction of pioneering drugs belonging to the aforementioned classes and all the drugs based on the different strategies are summarised in Figure 2 [20].

**Figure 3:**
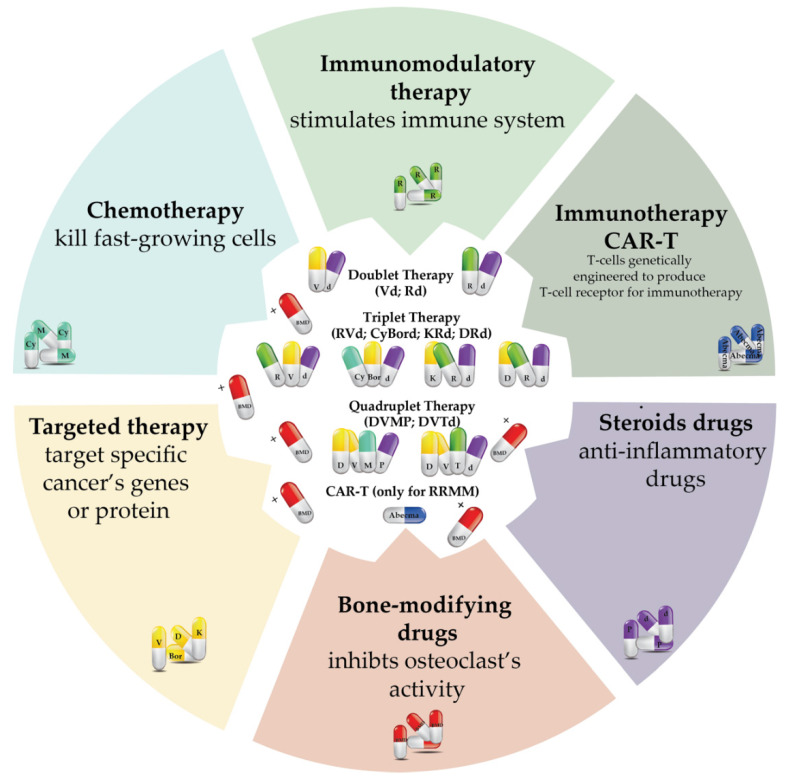
Most common combined and approved regimens for treatment of multiple myeloma. Drugs with a different mechanism of action can be used synergistically, resulting in duplet, triplet, or quadruplet therapy depending on the number of used combined drugs. Therapies are named using the first letter of the commercial drug name or active ingredient. Bone-modifying drugs are used before or after treatments to relieve bone pain. Doublet therapy: Vd (velcade–dexamethasone)/Rd (Revlimid–dexamethasone); triplet therapy: RVd (Revlimid–Velcade–dexamethasone)/CyBord (cytoxan–bortezomib–dexamethasone)/KRd (Kyprolis–Revlimid–dexamethasone)/DRd (Darzalex–Revlimid–dexamethasone); quadruplet therapy: DVMP (Darzalex–Velcade–melphalan–prednisone); DVTd (Darzalex–Velcade–Thalidomid–dexamethasone); immunotherapy: CAR-T (Abecma**^TM^**, only for RRMM); bone-modifying drugs (BMD) (bisphosphonates; monoclonal antibody denosumab).

As knowledge on myeloma increases, these drugs are no more intended as single drugs to be used separately, but rather as drugs that may work together to globally implement myeloma treatment. They can be used synergistically, resulting in doublet therapy (two compounds), triplet therapy (three compounds), or quadruplet therapy (four compounds) (Figure 3). Although different regimens are currently used, and new ones are under investigation, to our knowledge, the most used frontline combined therapies are bortezomib–dexamethasone (Vd), lenalomide–dexamethasone (Rd), cyclophosphamide–bortezomib–dexamethasone (CyBord), lenalomide–bortezomib–dexamethasone (RVd) [21,22], bortezomib–thalidomide–dexamethasone (VTd), carfilozomib–lenalomide–dexamethasone (KRd), daratumumab–lenalomide–dexamethasone (DRd), daratumumab–bortezomib–melphalan–prednisone (DVMP), and daratumumab–bortezomib–thalidomide–dexamethasone (DVTd) (Figure 3).

Current quadruplet therapy DVTd appears to be very promising in the overall survival of newly diagnosed myeloma patients. Randomised, open-label, Phase 3 study CASSIOPEIA showed that the incorporation of daratumumab (anti-CD38mAb) in triplet regimen VTd before and after autologous stem-cell transplantation decreases cancer progression and death compared to the VTd regimen alone. In the last Phase 3 trial (NCT02541383), the death of two patients was reported to be a consequence of septic shock and natural killer cell lymphoblastic lymphoma [23]. In addition, other adverse effects such as neutropenia, lymphopenia, and stomatitis were identified [23,24,25].

Concerning the high frequency of both refractory and relapsed patients, a very innovative approach is treating RRMM with the leading chimeric antigen receptor therapy (CAR-T) [26].

CAR-T technology takes advantage of modified T-cell function that can more efficiently target myeloma cells in the patient’s body. Blood is collected from the patient or healthy donor, and isolated T cells are then manipulated into expressing chimeric antigen receptors on their surface. This new cell population is expanded and infused into the patient. Cells collected from patients can overcome a graft-versus-host response [27] because the patients receive their own manipulated cells. Still, they expand less efficiently than the healthy donor ones do, and this must be taken into account when this strategy needs to be used.

Once CAR-T cells are infused, the chimeric antigen receptor recognises myeloma cells, destroying them. There are several (completed, recruiting, or not yet recruiting) CAR-T clinical trials, alone or in combination with other drugs, for the different populations of patients who had developed either relapse or resistance to the aforementioned drug treatment [28]. Abecma**^TM^**, the commercial name for idecabtagene vicleucel (Ide-Cel), is one of the first B-cell maturation antigen (BCMA)-directed chimeric antigen receptor (CAR) T cells approved by the US Food and Drug Administration (FDA). Clinical-trial data show significant improvement in the treatment of RRMM patients [29,30,31].

Unfortunately, even though the data on the efficacy are promising for almost all clinical trials, several adverse effects have been identified (e.g., cytokine release syndrome, haemophagocytic lymphohistiocytosis/macrophage activation syndrome (HLH/MAS), brain-related side effects (neurotoxicity), and prolonged cytopenia) [32]. For these reasons, Abecma^TM^ has only been approved for relapsed or refractory patients to four or more frontline regimens containing at least one immunomodulatory agent, a proteasome inhibitor, and an anti-CD38 monoclonal antibody (Figure 3).

Lastly, bone-modifying drugs (bisphosphonates or denosumab) are often used to inhibit bones’ osteoclast activity from treating, and to relieve bone pain and fractures that typically affect myeloma patients, and they can be used in combination with other drugs or alone [33] (Figure 3).

## 3. Marine-Derived Compounds Commercial Approved and in Clinical Trials

The literature shows that there has been an increasing interest in marine natural products in the last few decades. After 2000, scientific articles on MNPs rapidly increased thanks to the advent of new leading technologies that shed light on marine species’ peculiar diversity. Searching in PubMed by using the key phrase “marine natural products “from 1919 to 2000 (81 years), there are almost 9 papers per year, while from 2000 to 1 May 2022 (nearly 22 years), there has been a boost of about 538 articles for year (of which 1600 were published only in 2021). The root of the market for MNPs is about 20 years, passing through marine organisms sampling to the different clinical trials. Other bioactivity screening and chemical identification pipelines may be used. A study can start by selecting or sampling the species to be tested, bioactivity screening species pools, or with direct chemical extraction and metabolite analyses.

As reviewed by Martínez Andrade et al. (2018) and others [34,35,36,37], several studies showed that marine organisms may produce bioactive compounds with cytotoxic activity and antitumoural properties [34,35,36,37].

Some marine-derived natural compounds isolated from sponges, molluscs, cyanobacteria, corals, or tunicates have been commercially approved for MM by the US or Australian FDA and the European Medicines Agency (EMA) (in bold in Table 1; https://www.midwestern.edu/departments/marinepharmacology/clinical-pipeline; accessed on 1 May 2022). Others are currently under clinical trial (in bold, Table 2) or preclinical study investigations (Table 3).

Table 1 and Table 2 also list drugs in use and clinical trials for other tumours to give a complete overview of marine-derived compounds used in cancers. These drugs could be further studied for different applications. For example, clinical trials are evaluating the possible use of cytarabine, approved for leukaemia therapy and used in a BEAM combination regimen (carmustine, BCNU/etoposide/cytarabine/melphalan) in MM patients [38].

Among commercially approved compounds, just a couple are used in MM patients (in bold in Table 1). Belantamab–mafodotin, derived from a mollusc/cyanobacterium (https://www.midwestern.edu/departments/marinepharmacology/clinical-pipeline; accessed on 1 May 2022), is a monoclonal antibody conjugated to cytotoxic agent monomethyl auristatin F (MMAF). After belantamab–mafodotin internalisation by the tumour cells, the cytotoxic agent is released, leading to apoptosis [39]. The antibody intensifies the recruitment and activation of effector immune cells, which kill tumour cells through cellular cytotoxicity and phagocytosis. BLENREP**^TM^**, manufactured by GlaxoSmithKline, was approved by US FDA in 2020 and EMA for the treatment of adult patients affected by multiple myeloma, and it has resulted in relapsed or refractory patients to at least five different conventional therapies. 

The second approved compound was plitidepsin (Aplidin^®^) [40,41]. Plitidepsin is a marine-derived anticancer compound isolated from Mediterranean tunicate *Aplidium albicans*. It has been approved after several clinical trials on myeloma patients alone or in combination with other drugs (Clinical Trials identifiers: NCT00229203, NCT01102426, NCT03117361, NCT02100657). This cytotoxic peptide interacts with elongation factor 1-alpha 2 (eEF1A2), which is overexpressed in myeloma cells, leading to a proapoptotic event [42]. Plitidepsin (Aplidin^®^, PharmaMar) has been approved in some countries combined with dexamethasone to treat patients becoming pentaresistant to the above-mentioned conventional treatments [43]. Only Australia approved its use in 2018, while the US FDA [44] and EMA [45] refused to approve the use of this drug because of several side effects. Very recently, the European General Court cancelled the EMA decision, reverting the marketing authorisation application for Aplidin^®^ [46].

While a few drugs have been approved for myeloma patients, others are currently under several clinical trial investigations (Table 2). As previously discussed, the incidence of MM is increasing, and things are much more complicated if we consider not only new patients, and refractory and relapsed ones for whom available therapies failed, but also the several adverse events caused by therapies that impact the life quality of patients. Adverse effects can vary depending on treatments and the patient’s age [23,24,47]. The scenario can be even worse considering that frontline therapies are a combination of different drugs with peculiar side effects. The most common are drowsiness, dizziness, constipation, skin rash, neutropenia, and peripheral neuropathy. Two strategies are taken to overcome these with uncomfortable side effects. One is the post-therapy management of fragile patients, especially relapsed and refractory ones (RR) [47]; the other is to increase the efforts across the scientific community in the discovery of new compounds with specific features, such as the ones with immunomodulatory activity or compounds able to target cancer cells. For this reason, research on MNPs is attracting interest.

**Table 2:** Marine-derived drugs under clinical trial evaluation. Commercial name (when available), manufacturing company, clinical trial phase, cancer type and clinical trials identifier [48] are reported for each compound. Compounds in clinical trials for MM are in bold. NSCLC, nonsmall-cell lung carcinoma; SCLC, small-cell lung carcinoma; MM, multiple myeloma; RRMM, relapsed refractory multiple myeloma; DLBCL, diffuse large B-cell lymphoma), S.T., solid tumours; GBM, glioblastoma; B.C., breast cancer; RCC, renal-cell carcinoma; PNET, primitive neuroectodermal tumour; EOE, extraosseous Ewing’s sarcoma; N/A, not available.

Compound	Marine Organism	Commercial Name	Company	Clinical Trial Phase	Cancer Type	Clinical Trials Identifier
**Salinosporamide-A**	Marine actinomycete	**Marizomib** ^®^	Triphase	Phase II Phase I Phase III Phase II	MM, RRMM GBM GBM GBM	NCT05050305 NCT04341311 NCT03345095 NCT03463265
**Plinabulin** ^®^ **(NPI-2358)**	Fungus	N/A	BeyondSpring Pharmaceuticals	Phase II Phase II Phase III Phase I Phase II	MM, NSCLC, NSCLC, NSCLC, SCLC	NCT05130827 NCT02846792 NCT02504489 NCT02812667 NCT03575793
Lurbinectedin (PM01183)	Tunicate	Zepzelka^TM^	PharmaMar	Phase II Phase I Phase I Phase III Phase III Phase VI Phase I Phase I Phase I Phase I Phase I Phase II	SCLC SCLC SCLC SCLC SCLC SCLC ST ST ST STST ST	NCT04358237 NCT04253145 NCT05244239 NCT05153239 NCT05091567 NCT04894591 NCT04638491 NCT05072106 NCT02611024 NCT05063318 NCT05101265 NCT05126433
AGS-16C3F	Mollusc/cyanobacterium	N/A	Agensys and Astellas Pharma	Phase II	RCC	NCT02639182
PM060184	Sponge	N/A	PharmaMar	Phase I Phase I	ST ST	NCT01299636 NCT02533674
Tisotumab vedotin	Mollusc/cyanobacterium	HuMax^®^-TF-ADC	GenMab	Phase II Phase II Phase II Phase II Phase II	ST ST Cervical cancer ST Cervical cancer	NCT02552121 NCT02001623 NCT03438396 NCT03245736 NCT03786081
Ladiratuzumab vedotin (SGN-LIV1A)	Mollusc/cyanobacterium	N/A	Seattle Genetics	Phase II Phase I Phase II	ST BC BC	NCT04032704 NCT01969643 NCT03310957
Telisotuzumab vedotin (ABBV-399)	Mollusc/cyanobacterium	N/A	Abbvie	Phase II Phase III Phase I	NSLC NSLC ST	NCT03539536 NCT04928846 NCT02099058
Enapotamab vedotin	Mollusc/cyanobacterium	HuMax^®^-AXL	Genmab	Phase II	ST	NCT02988817
RC-48	Mollusc/cyanobacterium	N/A	RemeGen	Phase II	BC,	NCT05134519
				Phase II	Melanoma Stage II, HER2-positive, advanced melanoma	NCT05135715
				Phase II	Gastric cancer	NCT05241899
				Phase II	Biliary tract cancer	NCT04329429
				Phase II	Muscle invasive Bladder carcinoma,	NCT05297552
				Phase II	NSCLC	NCT04311034
				Phase II	Urothelial carcinoma	NCT03809013
				Phase I	ST	NCT02881190
				Phase II	HER2-positive metastatic or unresectable urothelial cancer	NCT03507166
				Phase III	Gastric cancer	NCT04714190
				Phase II	Urothelial cancer	NCT04073602
				Phase III	HER2-positive metastatic breast cancer, breast diseases	NCT03500380
				Phase II	Gastric cancer	NCT03556345
				Phase II	Bladder cancer	NCT05016973
				Phase III	Breast cancer	NCT04400695
				Phase III	Urothelial cancer	NCT05302284
				Phase II	Gastric cancer	NCT05313906
				Phase II	Breast cancer	NCT03052634
				Phase II	Urothelial cancer	NCT04879329
				Phase II	Gastric cancer	NCT05113459
CAB-ROR2 (BA-3021)	Mollusc/cyanobacterium	N/A	BioAtla	Phase II	NSCLC, triple-negative breast cancer, melanoma, head and neck cancer	NCT03504488
CX-2029 (ABBV-2029)	Mollusc/cyanobacterium	N/A	AbbVie and CytomX Therapeutics	Phase II	ST, head and neck cancer, NSCLC, pancreatic cancer, DLBCL	NCT03543813
W0101	Mollusc/cyanobacterium	N/A	Pierre-Fabre	Phase II	Advanced or metastatic ST	NCT03316638
ARX-788	Mollusc/cyanobacterium	N/A	Ambrex and ZhejiangMedicine	Phase II	Breast cancer,	NCT04829604
				Phase I	ST, breast neoplasms, gastric neoplasm,	NCT03255070
				Phase II	HER2 mutation-related tumours HER2-amplified ST,	NCT05041972
				Phase II	Breast cancer	NCT05018676
				Phase II	HER2-positive, metastatic breast cancer,	NCT05018702
				Phase I	Breast neoplasms, stomach neoplasms,	NCT02512237
				Phase I	breast neoplasms	NCT04983121
XMT-1536	Mollusc/cyanobacterium	N/A	Mersana Therapeutics	Phase II	Platinum-resistant ovarian cancer, NSCLC	NCT03319628
				Phase II	Platinum-sensitive ovarian cancer (UPGRADE-A)	NCT04907968
				Phase II	Ovarian cancer, NSCLC	NCT04396340
ALT-P7	Mollusc/cyanobacterium	N/A	3SBio and Alteogen	Phase I	ST	NCT03281824
MORAb-202	Sponge	N/A	Eisai	Phase I Phase II	ST ST	NCT03386942 NCT04300556
PF-06804103	Mollusc/cyanobacterium	N/A	Pfizer and AbbVie	Phase I	Breast neoplasms	NCT03284723
ZW-49	Mollusc/cyanobacterium	N/A	Zymeworks and BeiGene	Phase I	HER2-expressing cancers	NCT03821233
Synthetic alkaloid	Tunicate/sponge	Zalypsis^®^ (PM00104)	PharmaMar	Phase II	Ewing’s Sarcoma, PNET, Askin’s Tumor of the chest wall, EOE	NCT01222767
				Phase II	Uterine cervical cancer, endometrial cancer	NCT00900562
				Phase I	ST lymphoma	NCT00359294

Among the several clinical trials of marine-derived compounds, most of them are used to treat solid tumours (glioblastoma, breast cancer, nonsmall- and small-cell lung cancer, and others). Marizomib^®^, manufactured by Celgene, is one of the marine-derived compounds enrolled in several clinical trials. Marizomib^®^, the commercial name for salinosporamide A, is an irreversible and potent proteasome inhibitor derived from a marine actinomycete, with distinct activity and specificity from those of other proteasome inhibitors [49]. A Phase 1 clinical trial (NCT00629473) established the safety and effectiveness of marizomib^®^ in patients with MM [50]. Other Phase 1 trials (NCT00461045; NCT02103335) determined the maximal tolerated dose of marizomib^®^ in patients affected by myeloma alone or in combination with FDA-approved immunomodulatory treatment such as Pomalyst^TM^ (pomalidomide), dexamethasone (LODEX^TM^) or frontline therapies such as Revlimid^TM^ (lenalidomide), and Velcade^TM^ [51,52]. These trials showed that marizomib^®^ is well-tolerated with minor side effects on other proteasome inhibitors treatments, having promising efficacy on relapsed and refractory myeloma patients. However, despite data on the activity of this drug, alone or in combination with other approved drugs, it has not yet been approved by FDA [53]. Another compound, named plinabulin^®^ and manufactured by BeyondSpring Pharmaceuticals, is the synthetic analogue of the diketopiperazine phenylahistin discovered from marine and terrestrial *Aspergillus* sp. Despite a preclinical study in 2011 showing the proapoptotic antimyeloma features of plinabulin [54], the plinabulin^®^ Phase II trial is instead investigating the combination of pinabulin^®^ and pegfilgrastim to efficiently reduce neutropenia, which is a common adverse event in patients affected by multiple myeloma after autologous hematopoietic cell transplantation.

## 4. Marine-Derived Compounds in Preclinical Study for MM

Various marine active compounds are under investigation in preclinical in vitro or in vivo studies for MM (Table 3).

**Table 3:** Marine-derived compounds or extracts with activity in vitro or in vivo. Preclinical studies showing marine-derived compounds with antimyeloma activity in vitro/in vivo. Mechanism of action (when reported), marine organism, and experimental conditions are reported for each compound. MM, multiple myeloma); UPS, ubiquitin–proteasome system; N/A, not available.

Compound	Marine Organism	In Vitro/In Vivo	IC50 or Tested Concentration	Administration	Mechanism of Action	Refs
Zalypsis^®^ (synthetic alkaloid)	Tunicate/Sponge	In vitro Ex vivo	0.1–50 nM 3 × 3-1 mg/kg once weekly	In cell culture medium Intravenous	Induce apoptosis due to DNA breaks	[55]
		Phase I/II	3 × 2.0 mg/m^2^ Every 28d	Intravenous		[56]
		In vivo (CB17-SCID mice)	0.75 mg/kg weekly, for three doses in combination with bortezomib and dexamethasone	Intravenous		[57]
Sarcophytonin-A, sarcophytoxide, sarcophine, laevigatol-A	Soft corals	In vitro (HEK293T cells transiently expressing EGFP-UL76)	1–25 μg/mL	In cell culture media	UPS Inhibition	[58]
Reniochalistatin-E	Sponge	In vitro (MM cell line RPMI-2886)	IC50 values of 4.9 μM	In cell culture media	N/A	[59]
Nocardiotide-A	Actinomycetes/ sponge	In vitro (MM cell line MM.1S)	IC50 values of 8, 11, 12 μM/mL	In cell culture media	N/A	[60]
Lehualide-B	Sponge	In vitro (MM cell lines NCI H929, U266, and RPMI-8266)	0.1−10 μM	In cell culture media	Inhibition of mitochondrial complex I	[61]
Smenamide-A	Sponge	In vitro (MM cell line SKM-M1 and RPMI-8226)	50 and 100 nM, 1 and 5 μM	In cell culture media	N/A	[62,63]
Smenospongidine	Sponge	In vitro (MM cell line RPMI-8226)	10–20 μM	In cell culture media	B-catenin downregulation	[64]

Some of them are reported in Table 3, such as reniochalistatin-E, containing proline-rich cyclic peptides isolated from marine sponge *Reniochalina stalagmitis* [59]; nocardiotide-A, a cyclic hexapeptide isolated from the broth culture of *Nocardiopsis* sp. associated with marine sponge *Callyspongia* sp. [60]; and smenamide-A, isolated from Caribbean sponge *Smenospongia aurea* [65]. Cytotoxic activities were evaluated by using in vitro cytotoxic assays such as MTS (3-(4,5-dimethylthiazol-2-yl)-5-(3-carboxymethoxyphenyl)-2-(4-sulfophenyl)-2H-tetrazolium) [59,62,63] or MTT (3-(4,5-dimethylthiazol-2-yl)-2,5-diphenyltetrazolium bromide) [60], while proapoptotic features were evaluated by cytometric analysis using a fluorescein isothiocyanate (FITC) Annexin V Apoptosis Detection kit [62]. In addition, the synthetic analogous of the active part of the smenamide-A was produced to further investigate the possible MM antiproliferative activity in vitro [62]. Unfortunately, except for reniochalistatin-E, for which less cytotoxicity has been reported in other tumour cell lines, giving an approximative indication of the specificity of this compound, nocardiotide-A and smenamide-A cytotoxic and proapoptotic properties are shared in vitro experiment with other cancers cell lines, and have not been tested on cells derived from healthy donors. Further investigations are needed to characterise the molecular mechanisms of action of the above-mentioned marine compounds and their cancer specificity. More is known for the remaining marine products. Steiner et al. screened different compounds and compared them to Aplidin^®^ and bortezomib, used as golden standards. Through multiple in vitro, in vivo, or ex vivo approaches, they identified at least three compounds (Zalypsis^®^, PM00113, and thiocoraline A) with potent activity in inhibiting myeloma expansion [66]. Cell viability was assessed in vitro on MM cell lines by flow cytometry. Specific antimyeloma activity has been characterised on human MM cell line stable expressing green fluorescent protein (eGFP), and cocultured with primary human bone marrow mesenchymal cells plus collagen type I as the extracellular matrix environment. Tumour growth was controlled during treatments using the MM xenograft model generated by grafting the above-mentioned cocultured MM eGFP cell lines on chicken embryos.

Moreover, the antiangiogenic properties of the best-performing compounds were tested and confirmed using an in vivo chick chorioallantoic membrane (CAM) assay [66]. In particular, Zalypsis^®^ has been investigated in other independent studies underlying the molecular mechanism of action and confirming the efficient inhibition of myeloma growth [55,56,57]. Ocio and colleagues showed that the antimyeloma activity of Zalypsis^®^ is due to the intrinsic properties of this compound to exert double-strand DNA breaks after inducing histone-H2AX phosphorylation and p53 overexpression, leading to the apoptosis of MM cells in a specific manner [55]. They investigated cytotoxicity on two MM cell lines, MM1R and RPMI-LR5, selected for their resistance to conventional antimyeloma treatments. All cell lines were sensitive to Zalypsis^®^ treatment. It was tested for cytotoxicity on nontumour CD34+ cells from two donors, and one patient with MM. Zalypsis^®^ was less cytotoxic in CD34+ than it was in tumour cells. Its synergistic effect with other drugs used against myeloma has also been evaluated with favourable outcomes, thus suggesting the possibility of using it in combination with dexamethasone, doxorubicin, melphalan, and lenalidomide [55]. In the in vivo studies, Zalypsis^®^ inhibited the growth of MM1S and OPM-1 xenograft plasmacytomas in mice, and appeared to be well-tolerated [55]. A study in 2016 confirmed the mechanism of action proposed for Zalypsis^®^ in patients with RRMM [56]. In another study in 2017, Lopez-Iglesias et al. reported the preclinical evaluation of the combination of Zalypsis^®^, bortezomib, and dexamethasone (ZaBDe). This combination provoked a synergistic DNA-damaging effect in MM cells, and decreased NF-kb translocation into the nucleus [57]. To evaluate the synergy in a physiological context, they used both in vivo and ex vivo experiments [57], confirming the capability of Zalypsis^®^ to inhibit tumour growth in the triplet combination. As what frequently happens in this kind of preclinical studies, several adverse effects were found. Body weight loss was observed in all mice that had received combinations that included Zalypsis^®^; 2 of the 10 mice treated with the ZaBDe combination died from the toxicity [57]. However, the eight surviving mice in the ZaBDe group almost completely regained their initial body weight, indicating that dose adjustment, duration, and combination therapy must be further studied to enter into clinical trials. For PM00113 and thiocoraline A, no update studies are reported. Still, it would be reasonable to invest in these compounds, as they belong to the same screening as that where Zalypsis^®^ was characterised [66].

Another very useful screening assay was set up by Ling and colleagues [58] to discover new drugs that inhibit the ubiquitin–proteasome system (UPS). Targeting the UPS is a common strategy to treat myeloma cancer, and the most used drugs are bortezomib, carfilzomib, and ixazomib. They used cell-based high-content measurements of EGFP-UL76 aggresomes to test several compounds, and identify those that affect the UPS pathway [58]. Thanks to the assay that they had established, they were able to identify at least four active compounds (sarcophytonin A, sarcophytoxide, sarcophine, and laevigatol A) extracted from soft corals. These compounds efficiently inhibit the UPS, similar to the most famous and used inhibitors bortezomib and MG132 [58]. In addition, smenospongidine isolated from marine sponge *Smenospongia* sp. works as an antagonist of the Wnt/β-catenin signalling pathway [64]. This activity led to the degradation of upregulated intracellular β-catenin, a critical pathological feature in MM progression [67]. Wnt signalling in MM is activated by stromal cells in the bone marrow microenvironment secreting Wnt ligands, and regulated by transcription regulator catenin.

Furthermore, the Wnt/-catenin pathway promotes osteoblast development, leading to bone formation. Its dysregulation is linked to MM cell proliferation and treatment resistance [68,69]. Further investigations need to confirm whether smenospongidine is a good candidate for a new therapy to fight MM progression.

Marine natural product lehualide B from Hawaiian and Tongan marine sponges (*Plakortis* sp.), and its synthetic version showed very potent antimyeloma activity. The cytotoxic activity of lehualide B has been deeply investigated in several myeloma cell lines using an MTT assay. In particular, antimyeloma activity is exerted through the selective inhibition of mitochondrial complex I, which results in the specific inhibition of myeloma progression [61], as MM cells are susceptible to mitochondrial inhibitors [70].

## 5. Discussion

Despite the increasing knowledge of MM features from the literature, and the advancement of cutting-edge technologies, especially regarding drug discovery, multiple myeloma remains an incurable disease. Besides the benefits of the current treatment for patients affected by multiple myeloma, the several severe adverse effects that occur during and after treatments affect the life quality of patients. Among many side effects, the most common are thrombocytopenia and neutropenia, neuropathy, and fatigue. In some cases, adding a new generation of approved drugs such as proteasome inhibitors, immunomodulatory agents, and monoclonal antibodies to first-line therapy can even worsen these side effects [71]. The addition of daratumumab or ixazomib to a lenalidomide and dexamethasone regimen increases the frequency of Grades 3 and 4 neutropenia from 37% to 52%, and the frequency of Grades 3 and 4 thrombocytopenia from 9% to 19% respectively [25,72]. The side effects are the primary reason for which new drugs, either from natural sources or not, did not receive final commercial approval (e.g., natural marine product Aplidin^®^) or, as it happens for recent drugs (e.g., CAR-T/Abecma**^TM^** and the new conjugated natural marine product MAb/BLENREP^TM^), only approved for use in MM patients who had relapsed with at least three or five approved drugs. On the other hand, it is indisputable that the use of new generations of drugs such as MAb combined with conventional therapies is increasingly transforming MM into a chronic disease, effectively increasing patient survival. For these reasons, the scientific community is continuously looking for new compounds or the modifications of lead compounds with the same or more efficacy and fewer adverse effects. Compounds derived from marine environments exert great cytotoxic activity against cancer cells [34]. Considering that only a small fraction of marine-derived compounds has been tested in vitro or in vivo, the road to the market appears to be quite long. It is necessary to undertake more efforts in identifying new MNPs by also using appropriate screening to identify suitable targets for each specific pathology. New drugs that are most successful in fighting MM are part of drug classes with specific targets, such as proteasome inhibitors or cell-targeted therapies such as conjugated antibodies that recognise specific cell types. Regarding the chemical structures, bioactive compounds are very variable, ranging from cyclic peptides to alkaloids, confirming the huge chemical diversity available in the marine environments that can exert bioactivity. MM is a remarkably complex cancer, and MNPs for MM are hardly and slowly identified. Without a collective effort by the whole scientific community, MM may remain an incurable disease for a long time. In addition, there are at least three more good reasons to invest in MNPs: a very high level of species diversity and compound heterogeneity, ecosustainable sources (e.g., easily cultivable microorganisms), and versatile applications. For example, in a recently published short communication, diatoms were functionalised with bisphosphonates to create an innovative biomaterial to treat a broad spectrum of bone degradation disorders such as osteoporosis, Paget’s syndrome, and bone cancers including myeloma [73]. With the advent of omics technologies and genetic engineering, researchers are also able to create a modified organism (e.g., bacteria and microalgae) able to produce an increased amount of the compounds of interest or transfer the production of the compound of interest into a host (heterologous expression), as was discussed by Lauritano et al. [74].

Furthermore, marine bacteria and microalgae can be cultured in controlled conditions in photobioreactors, obtaining huge biomass without damaging the environment. Therefore, they represent a rich and ecosustainable source of novel compounds with anticancer properties [34]. Many drugs can be developed, starting from marine sources in the future, if the MNP discovery pipeline becomes faster and more efficient. Considering these aspects, we wish to increase the scientific community’s attention to MNP drug discovery, especially in the cancer field, providing new possible molecules for myeloma therapy and other applications.

## 6. Conclusions

Multiple myeloma is a very complex cancer for which no cure is currently available. The intuition and effort of the scientific community in identifying and combining new drugs that act in different ways made possible to increase the patient survival. However, the second generation of refractory and recurrent patients (RRMM) is generating an even more complex challenge. Compounds of marine origin have shown over the last few decades to have excellent anti-tumour properties. However, of these compounds, only a tiny percentage have reached clinical trials, and even less the market. In addition, only a small amount of the marine compounds have been tested for multiple myeloma until now. The reason for this low success lies partly in the complexity and length of the pipeline that goes from MNPs discovery to application in humans. This review aimed to provide clues to the enormous potential of marine products in treating multiple myeloma, suggesting and hoping that greater attention from the scientific community in this regard could shorten the pipeline time and make this process faster and more efficient.

## Figures and Tables

**Table 1:** Approved marine drugs for cancer therapy. Active ingredients are actively used to treat MM (in bold) or other cancer types. Derived marine organism, commercial name, manufacturing company, and cancer types are reported for each active ingredient. NSCLC, nonsmall-cell lung carcinoma; SCLC, small-cell lung carcinoma; MM, multiple myeloma; and RRMM, relapsed refractory multiple myeloma.

Compound	Marine Organism	Commercial Name	Company	Cancer Type
**Belantama-mafodotin**	Mollusc/cyanobacterium	**BLENREP^TM^** (2020) *	GlaxoSmithKline	RRMM
**Plitidepsin**	Tunicate: *Aplidium albicans*	Aplidin^®^ **	PharmaMar	MM, leukaemia, lymphoma
Lurbinectedin	Tunicate	Zepzelca^TM^ (2020) *	PharmaMar	Metastatic SCLC
Enfortumab vedotin—ejfv	Mollusc/cyanobacterium	PADCEV^TM^ (2019) *	Astellas Pharma and Seattle Genetics	Metastatic urothelial cancer
Polatuzumab vedotin (DCDS-4501A)	Mollusc/cyanobacterium	Polivy^TM^ (2019) *	Genetech/Roche	Non-Hodgkin lymphoma, chronic lymphocytic leukaemia, lymphoma, B-cell lymphoma
Trabectedin (ET-743)	Tunicate	Yondelis^®^ (2015) *	PharmaMar	Soft tissue sarcoma and ovarian cancer
Brentuximab vedotin (SGN-35)	Mollusc/cyanobacterium	Adcetris^®^ (2011) *	Seattle Genetics	Anaplastic large T-cell systemic malignant lymphoma, Hodgkin’s disease
Eribulin Mesylate (E7389)	Sponge	Halaven^®^ (2010) *	Eisai Inc.	Metastatic breast cancer
Cytarabine (Ara-C)	Sponge	Cytosar-U^®^ (1969) *	Pfizer	Leukaemia

* FDA-approved. ** Australia-approved, December 2018.
